# ‘Superfoods’: Reliability of the Information for Consumers Available on the Web

**DOI:** 10.3390/foods12030546

**Published:** 2023-01-26

**Authors:** Ángel Cobos, Olga Díaz

**Affiliations:** Departamento de Química Analítica, Nutrición y Bromatología, Facultade de Ciencias, Campus de Lugo, Universidade de Santiago de Compostela, 27002 Lugo, Spain

**Keywords:** superfoods, consumers, online information, disease prevention, health

## Abstract

The term ‘superfoods’, used frequently with marketing purposes, is usually associated with foodstuffs with beneficial health properties. ‘Superfoods’ appears in many information sources, including digital media. The information they provide is easily accessible for consumers through Internet search engines. The objective of this work is to investigate the data that web pages offer to consumers and their accuracy according to current scientific knowledge. The two main search engines were utilized for English language websites search, introducing the term ‘superfoods’. In total, 124 search results were found. After applying the selection criteria, 45 web pages were studied. A total of 136 foods were considered as ‘superfoods’ by sites; 10 of them (kale, spinach, salmon, blueberries, avocado, chia, walnuts, beans, fermented milks and garlic) were mentioned on at least 15 sites. Nutritional and healthy properties displayed on sites were compared to scientific information. In conclusion, websites present the information in a very simplified manner and it is generally not wrong. However, they should offer to consumers comprehensible information without raising false expectations regarding health benefits. In any case, ‘superfoods’ consumption can have salutary effects as part of a balanced diet.

## 1. Introduction

The term ‘superfood’ was introduced in the 1990s to indicate a special category of food with superior nutritional properties [1]. There are many definitions of ‘superfood’, but it usually names foodstuffs characterized by their richness in macro- and micronutrients which have positive effects on human health and are useful for illness prevention [2]. Besides, these properties are inherent to the product (without any addition). In the concept of ‘superfoods’ is commonly included the idea that they are naturally produced and, frequently, are associated with indigenous populations and traditional agricultural practices [3]. However, in many cases, these foodstuffs are produced far from the developed regions of the world, and the increase of their demand has consequences in producer countries, such as the displacement of other traditional crops by monoculture and the change from traditional to intensive agricultural practices [1,3].

In fact, there is not a clear and legal definition of ‘superfoods’; nowadays, many foodstuffs are included in this group by the marketing of the food industry. The term is commonly used in informal communication, on social media and by popular influencers, which increases the popularity of a wide variety of foods [4]. Although the scientific literature endorses the health benefits of ‘superfoods’, other considerations must be contemplated, such as the factors that influence the chemical composition of foods and the potential health risks of consumption of some ‘superfoods’ [5].

Searching for nutrition information has changed in recent decades due to the increase of the use of technologies for communication at the expense of traditional sources [6]. Search engines are frequently preferred for seeking health information for their convenience and the capability of providing information from multiple sources [7]. People can obtain information rapidly about food through digital technologies (blogs, online lifestyle magazines, cooking web pages, etc.), which has modified the concept of the expert. These facts have made much easier the expansion of misinformation around foodstuffs and nutrition; social media, food influencers or news media do not always provide evidence-based or accurate information [6,8].

The classification of foodstuffs in the category of ‘superfoods’ could affect the demand, the consumption and the attitudes of consumers towards them; many studies related to these effects can be found [2,3,5,9]. Moreover, a great attention has been paid in recent years to the new digital sources that people use for getting information about health, nutrition and food [8,10,11,12].

Nevertheless, to the best of our knowledge, there are no studies about the accuracy of the information that consumers can find on the Web related to the composition and health benefits of ‘superfoods’. In this regard, the overall objective of this work is to explore the information about ‘superfoods’ that web pages offer and which consumers can find by using search engines. In order to achieve this aim, it was investigated which are the most frequently mentioned ‘superfoods’ on websites, the claims about nutrients and beneficial health effects that they report and finally, the reliability of this information according to the data reported in recent scientific literature.

## 2. Materials and Methods

A Websites search was carried out using two search engines, Google and Bing; according to FrontPage Data and Backlinko [13], Google is the most commonly used site, by 94% of searchers, while Bing is second, with a share of 2.7%. The computer used in the study was previously cleaned of cookies and trackers applying Spybot Search and Destroy software (version 2.8.67.0; Safer Networking Ltd., Greystone, Ireland).

Due to preset profiles created by search engines and websites in previous searches performed on the computer used in the study, the Opera browser and its free virtual private network were used in order to avoid any bias. Furthermore, the identification of the computer’s IP address location was averted, making it possible to get results without any local bias. Another search engine, Startpage, was also used for the purpose of verifying that, in the case of Google search, the responses were free of distortion due to previous profiles associated with the computer’s IP address. Startpage delivers Google search results with additional personal data protection that avoids profile creation.

The term searched was ‘superfoods’ and search engines were configurated to give results only in English. Configuration settings in Google were only English pages published in any region, while in Bing, it was necessary to select the United Kingdom (a European location was preferred) to obtain results only in English. In both cases, searches were carried out on 20 May 2022, recovering results corresponding to two periods of time, any time and the past year.

The results considered in the study were those found in the first and the second page of the search engines, due to only 1.5% people clicking on the second Google search page or further when looking for terms related to health topics [13].

**Figure 1:**
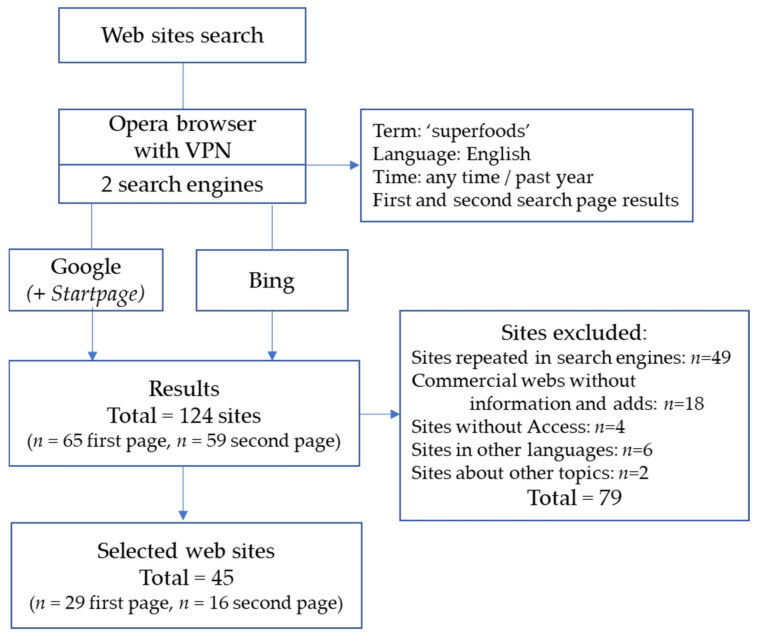
Flow diagram of the websites search and selection process. VPN: virtual private network.

A total of 124 search results were found by using the three search engines, 65 in the first search pages and 59 in the second ones. Similar figures were found for the two periods of time: 65 results for ‘any time’ and 59 for ‘past year’. The results obtained using Startpage were the same as those achieved with Google, although they gave two different, so the responses were not affected by prior search profiles. The selection of the search results was carried out using the following criteria: purely commercial websites without any information, adds, sites repeated in the same or in different search engines, pages without access, in languages other than English or about other topics were removed. Finally, 45 web pages were selected (29 and 16 in the first and in the second search page, respectively). Figure 1 shows the flow diagram of the websites search. The URLs of the selected web pages can be found as supplementary materials (Tables S1 and S2).

**Figure 2:**
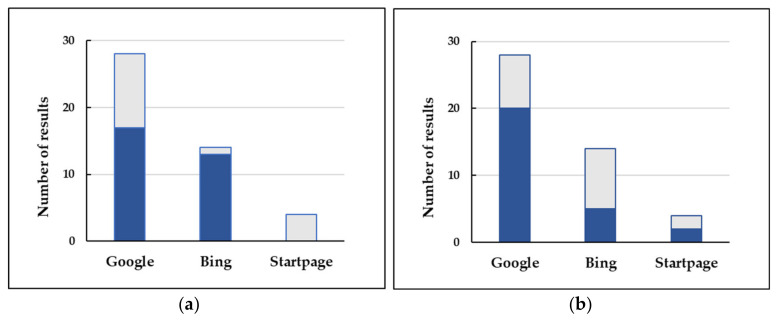
Number of selected web pages found using the three search engines: (**a**) On the first (blue) and the second search page (grey); (**b**) Per period of time: any time (blue) and past year (grey).

Figure 2 shows the selected results obtained using the three search engines in the first and the second search page (Figure 2a) and the number of results per period of time (Figure 2b). Most ‘any time’ results corresponded to pages dated 2021 and 2022 (71.1% of the total).

**Figure 3:**
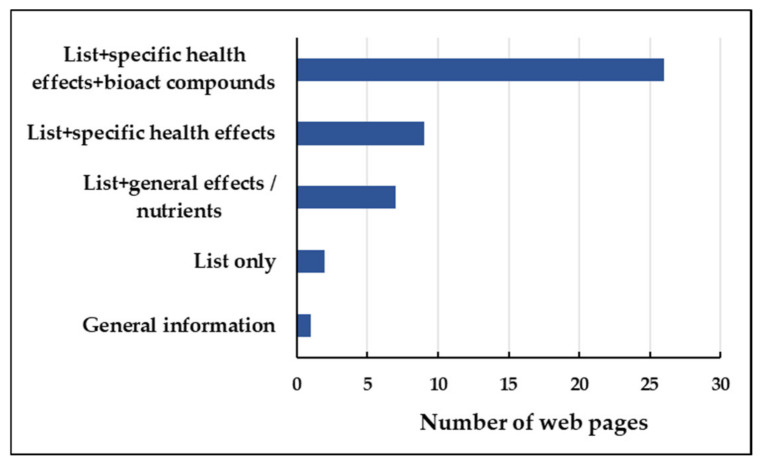
Number of selected web pages that provided different types of information.

Most of the websites selected, 20 corresponded to informative pages about health, medicine, wellness, sports for the general population; 7 to communication media (newspapers, magazines, television channels); 5 to blogs about beauty, recipes, sports; 5 to sites of shops with general information about foods; 5 to pages of medical centers, associations and organizations related to health; and 3 were sites of academic organizations (universities). Figure 3 shows the number of selected websites classified according to the type of information they provided. Eighteen websites gave references to scientific articles by direct links and/or indirectly by accessing to other web pages that contained the links. In total, 72.2% of websites that provided these references had updated their sources of information.

The scientific information about health benefits and nutrients of ‘superfoods’ necessary for the discussion was gathered using the Clarivate Web of Science database. The search was carried out using the names of each ‘superfood’ as keywords, limited to review articles published in the last five years and to the Web of Science categories of ‘Food Science and Technology’ and ‘Nutrition Dietetics’. The search concluded on 4 November 2022.

## 3. Results and Discussion

A total of 136 foods were considered as ‘superfoods’ by the selected sites. Table 1 shows the groups of ‘superfoods’ classified according to the number of times that the sites mentioned a food included in each of them. Each food was classified into a group according to the criteria used in many websites, generally based in some common characteristics and way of consumption.

The number of times that each food is named in the first and second page of the selected sites and the period of time corresponding to the information date of publishing in the sites (any time and past year) are shown in Table 2, Table 3, Table 4, Table 5, Table 6, Table 7, Table 8 and Table 9.

The most named group was the cruciferous vegetables that comprised twelve foods of the family *Brassicaceae* or *Cruciferae* (Table 2). Leafy greens constitute the second group, with five vegetables (Table 2). Fish and seafood results are displayed in Table 3. Fruits (Table 4) have been divided in two subgroups, berries (12 types) and other fruits (12 types), because berries were mentioned as a separate category of ‘superfoods’ in most websites, while the other fruits were named individually. Berries were the fourth most mentioned group of ‘superfoods’.

Eight whole grain cereals, eight seeds and three pseudocereals (Table 5) are grouped due to the most frequent way of consumption and the food products that can be manufactured with them as raw material. Bulgur wheat has been included in this list because it is recognized as a whole grain by the USDA, although it is made from cracked groats of several wheat species [14].

Nuts, legumes and fermented food groups results are displayed in Table 6. Fermented foods, a miscellaneous list, were frequently mentioned in sites as a group due to their arising from microbiological activity of the corresponding raw materials. Eight spices and herbs, three types of infusions and seven fats and oils were considered ‘superfoods’ (Table 7). Finally, Table 8 and Table 9 show the results for other plant-based foods (19 types) and for other animal-based (5 types) and other foods (water and nutritional yeast), respectively.

One food of each group, provided that it was mentioned by at least 15 sites, was selected to compare its properties, claimed by websites, with those supported by current scientific knowledge (Table 2, Table 3, Table 4, Table 5, Table 6, Table 7, Table 8 and Table 9). According to this, ten foods were studied: kale, spinach, salmon, blueberries, avocado, chia, walnuts, beans, fermented milks and garlic. Healthy and nutritional properties attributed to them are displayed in Table 10, Table 11 and Table 12. The health effects included in the list of properties have been selected according to those mentioned on websites.

### 3.1. Kale

Kale is the food most mentioned as a ‘superfood’ on websites (Table 2). This vegetable is member of the family *Brassicaceae* (previously named cruciferous, a term that is still frequently used in the literature) and belongs to the species *Brassica oleracea* var. *acephala*. Cruciferous vegetables have been studied in recent decades due to the association of diets rich in them with a decrease in the risk of several diseases; among them, kale has gained great popularity in the last years [15,16].

Table 10 and Table 11 show the nutrients of kale that were mentioned the most times by websites. It is considered as a good source of fiber, vitamins A, E, K and C, folates, calcium and iron. Compared with other plant foods, kale has a good concentration of fiber and vitamin K, is a moderate source of vitamin A and contains a higher concentration of vitamin C than other salad and cruciferous vegetables. Kale is also recognized as a food rich in other vitamins B (B_1_, B_2_, B_3_, B_6_) [15,17], although this was not found on selected sites.

Anti-nutritional factors in kale are not mentioned on sites. However, these compounds, which comprise oxalates, tannins and phytate, may interact with some nutrients and avoid their absorption [17]. Oxalates and phytate reduce the bioavailability of calcium, iron and other minerals; however, kale is low in oxalic acid compared with other vegetables, shows a high bioavailability of both calcium and iron, and is considered a good source of these minerals in diets for vegans [18].

According to the information provided by the sites, the main positive effects of kale consumption for health and disease prevention are its antioxidant activity, cardiovascular diseases protection, cancer risk reduction and prevention of digestive disorders (Table 12). In most cases, this information did not include the compounds or mechanisms involved in it. These effects match with the main biological activities associated with kale in scientific literature [15,19,20,21], although much information stems from *in vitro* experiments, while *in vivo* studies are limited.

The levels of phytochemicals and other nutrients in kale and other cruciferous vegetables, responsible for their health-promoting effects, depend on several factors, such as the variety, maturity stage of leaves, insect attacks, microorganism intrusion, location and environmental conditions of cultivation [16,19]. Besides, the content of bioactive compounds also changes due to post-harvest handling and cooking preparation. For these reasons, the positive effects of kale consumption on health could vary substantially. Apart from this, the beneficial health effects of cruciferous vegetables consumption are difficult to evaluate in general populations due to cultural factors, which affect the quantity consumed and make difficult the interpretation of epidemiological findings [20].

Antioxidant activity is related to vitamin C and E, polyphenols and carotenoids contents, and glucosinolates’ hydrolysis products. Regarding polyphenols, kale contains flavonoids, predominantly quercetin and kaempferol, and phenolic acids, mainly caffeic, ferulic and sinapinic acids. The red variety of kale also contains anthocyanins. The main carotenoids in kale are β-carotene, lutein, violaxanthin and neoxanthin, although their color is masked by chlorophyll. Glucosinolates and polyphenols also have anti-inflammatory activities [21].

Glucosinolates are sulfur-containing compounds that become bioactive when they are hydrolyzed by the endogenous enzyme myrosinase; this happens after the rupture of cellular membranes that allows the contact between enzyme and substrates, for example, by cutting during preparation or processing. This hydrolysis continues in the upper gastrointestinal tract after ingestion and also in the colon due to myrosinase-producing bacteria. The reaction products show bioactivity and include nitriles, thiocyanates and isothiocyanates, among others [22].

Antioxidant compounds contribute to health benefits in the prevention of chronic diseases, such as providing cardiovascular protection. The effect of kale consumption on the reduction of coronary artery disease risk has been confirmed by *in vivo* studies [15]. In general, cruciferous vegetables intake can confer important cardiovascular health benefits according to several epidemiological studies [23].

Cancer risk reduction associated with the consumption of *Brassica* vegetables has been observed in epidemiological studies and in dietary interventions, although studies about kale are sparse [24]. One of the compounds responsible for this effect is glucoraphanin, a glucosinolate that is converted to the biological active phytochemical sulforaphane by myrosinase [25]. Another glucosinolate, glucobrassicin, is transformed in indole-3-carbinol. Both compounds seem to have *in vitro* activity against prostate, breast, ovarian and colorectal cancer, and they can be found in kale [22,24].

Regarding digestive health, positive effects on inflammatory bowel disease, gastritis and gastric ulcer treatment of cruciferous vegetables intake (including kale) have been exploited by traditional medicine and demonstrated experimentally in laboratory animals. It may be explained by the increase of stomach pH, the stimulation of mucus synthesis and is also related to the effects of glucosinolate compounds as antimicrobials against *Helycobacter pylori* and the presence of prebiotic carbohydrates in kale, which contribute to balancing intestinal microbiota [15]. Other positive effects on health mentioned by a few sites that scientific information referred to are bone mass stabilization after menopause and prevention of neurodegenerative diseases [15,26]. Long-term consumption of diets rich in sulforaphane has also been cited as a promised approach to mitigate chronic inflammation states such as obesity, which also induces diseases like type 2 diabetes, among others [27].

Processing and cooking preparation methods could influence bioactive compounds and nutrients contents. Blanching and boiling cause significant losses in water-soluble nutrient components (vitamins, minerals, etc.) and phytochemicals (ascorbic acid, phenolic compounds, carotenoids) due to heat treatment and leaching. Glucosinolates can also leach to boiling water, so other methods, such as steaming or microwaving at a mild temperature, can preserve or even increase the conversion of glucosinolates to sulforaphane in cruciferous vegetables. Besides, thermal degradation of their metabolites, partial or total inactivation of myrosinase or washing method after cutting also modify the bioavailability of these compounds [25,28].

### 3.2. Spinach

Spinach (*Spinacia oleacia* L.) is the second most mentioned food on selected websites and the first leafy dark green vegetable (Table 2). The nutrients of spinach mentioned by websites are shown in Table 10 and Table 11. The compounds cited at least five times were fiber, vitamins A, K, C and folate, calcium and iron. Spinach is considered a good source of these compounds in the scientific literature. Besides, other nutrients that were taken account on a lower number of sites or on none of them, are there in considerable amounts; these are magnesium, manganese, potassium, phosphorus, zinc and copper [29,30,31]. Spinach contains some anti-nutritional compounds such as oxalates and phytic acid, which can reduce the bioavailability of some minerals such as calcium and iron [30].

The main positive effects of spinach cited by them on health are cardiovascular diseases protection, cancer risk reduction, antioxidant activity and bone health preservation (Table 12). Spinach contains polyphenols, carotenoids and ascorbic acid and phytochemicals with antioxidant activity, which are related to these effects, although other green leafy vegetables show higher concentrations of these compounds [32]. In the polyphenols group, flavonoids play an important role in spinach antioxidant properties; the predominant compounds are forms of patuletin and spinacetin. Carotenoids β-carotene and lutein are present in spinach at higher concentrations than in cruciferous vegetables [29]. The antioxidant activity is related with many beneficial health effects of spinach compounds. Epidemiological information about the benefits of spinach consumption suggests that it is connected to breast, esophageal and colon cancer risk reduction. Spinach anti-cancer activity is attributed to glycoglycerolipids and glycolipids, that may inhibit neovascularization and mitosis of tumoral cells [33], but also to other compounds such as p-coumaric acid, carotenoids and chlorophylls [29].

Cardiovascular disease prevention reached the highest number of mentions as a positive effect of spinach consumption. Although many scientific studies have demonstrated a significant inverse association of leafy greens intake and cardiovascular diseases incidence, other works show an absence of correlation. Frequently, the studies do not report the serving size weight, and lifestyle and dietary factors are not considered [23]. Spinach compounds deemed to be responsible for the cardiovascular protective effect are antioxidants and nitrates. Nitrates are converted into nitrites by host reductases and salivary bacteria, which are afterward reduced to nitrogen oxide (NO) in the stomach and in the circulatory system [29,34]. NO decreases blood pressure, arterial stiffness and platelet aggregation, and enhances endothelial function [34]. Large intakes of spinach leaves or drink increase the NO synthesis and reduce blood pressure [35].

The nitrate content in green leafy greens is affected by the type, amount and duration of nitrogen fertilization, and also by genetic and environmental factors. The presence of a high amount of nitrate in spinach may imply some health risk. Nitrite can be transformed, under certain conditions, in nitrosamines, which seem to be involved in the development of some types of cancer. Besides, the formation of methemoglobin after reduction of nitrate to nitrite can cause hypoxemia in infants. Inadequate storage conditions also improve the microbial reductase activity and the conversion to nitrite. Avoiding excessive consumption and an adequate control of fertilizers application and of the storage conditions considerably reduce these risks and spinach intake is thus considered safe [29,30]. None of these risks is mentioned on the sites’ contents.

Some other health benefits related to spinach consumption that have been reported in scientific literature obtained a low number of mentions on websites. These are the antimicrobial activity, anti-inflammatory effects, digestive and eyes health protection, weight control capacity or diabetes risk reduction [29,33]. In most of the *in vivo* studies, only spinach leaf extracts were used.

As occurs with other plant-based foods, phytochemicals concentrations in spinach are influenced by many factors, such as environmental conditions, agronomic practices (fertilization), harvesting stage and storage conditions (temperature, duration) [29]. Their content is also affected by processing and cooking methods. Drying reduces the availability of ascorbic acid, carotenoids and flavonoids in spinach. As in cruciferous and other leafy vegetables, blanching and boiling reduce phytochemicals concentration due to leaching and heating. The most appropriate method to retain water-soluble bioactive compounds is steaming. Even the increase of the concentration of total polyphenols and flavonoids in spinach has been reported, probably due to cell walls breakdown and phenolic compounds release promotion caused by heating. Microwave cooking shows variable results depending on the phytochemical compound [32,36].

### 3.3. Salmon

Salmon (*Salmo salar*) was the third most named food on websites as a ‘superfood’ (Table 3). The positive effects of salmon consumption on health that were found on sites are shown in Table 12. Those that were mentioned more times are cardiovascular diseases protection, the presence of anti-inflammatory compounds and the improvement of memory and learning skills. These effects are mostly related to the polyunsaturated fatty acids (PUFA) content of salmon, mainly to long-chain n-3 PUFA eicosapentaenoic acid (EPA) and docosahexaenoic acid (DHA). EPA and DHA contents are highlighted on 25 sites (Table 10). Both compounds prevent endothelial dysfunctions, decrease blood viscosity and pressure, and have anti-inflammatory properties [37]. However, the favorable effect of fish consumption on cardiovascular disease prevention could be attributed to the interaction of several lipid compounds present in fish (carotenoids, vitamins A, E and D, n-3 PUFA-rich phospholipids and glycolipids) and not only to n-3 PUFA content [38]. The carotenoid astaxanthin, a red pigment of salmon flesh without properties of provitamin A, is able to protect against oxidative stress and inflammation, decreasing cardiovascular risk. Salmon also contains lutein and β-carotene [39].

The positive effects of n-3 PUFA are induced by a multitude of metabolic mechanisms, but also through their influence on intestinal microbiota [40]. Vitamin D and selenium improve the neuroprotective properties of n-3 EPA and DHA [37]; these nutrients are only mentioned in 3 and 4 websites, respectively (Table 10 and Table 11). Less mentioned beneficial effects by sites, such as cancer risk reduction, immunity improvement, neurodegenerative diseases prevention and antidepressant action, have also been reported as related to n-3 PUFAs fish intake [41].

The second most mentioned nutrient by sites was protein. Fish proteins contain essential, nonessential and functional amino acids; the latter seems to be a great potential in the prevention of diabetes, cardiovascular diseases and neurological dysfunction, among other disorders [37]. The Vitamin E content in fish is higher than that of meat from terrestrial animals; this compound also has beneficial effects on cardiovascular diseases, some neurological disorders and immune function, and possesses antioxidant and anti-inflammatory properties [37,41]. However, it is not cited as an important nutrient in salmon by any sites (Table 11).

The amount and quality of n-3 PUFA and other fish lipid bioactives depend on the season, the fish development cycle moment, gender, way of living (farmed or wild), temperature and feed composition, and thermal treatments applied in processing and cooking [38]. PUFA composition is heavily affected by diet composition; in the last 15 years, farmed salmon EPA and DHA contents have decreased owing to the low availability of fish meal and fish oil and their substitution by vegetable oils in commercial feeds. Most of these oils show n-6/n-3 ratios much higher than those of fish oils and none of them contain EPA and DHA; although an increase of the relative retention of these fatty acids has been observed when fish oils are replaced by vegetable oils in feed, it is not enough to keep the levels in the salmon muscle. In 2015, the amount of these fatty acids was slightly lower in farmed salmon than in wild salmon; despite this, farmed salmon remains a good source of these compounds in the human diet [42]. Algal biomasses are currently used in commercial salmon feeds in order to improve the content of long-chain n-3 PUFA [43]. Nevertheless, other factors modify the incorporation of these fatty acids into fish tissue, such as type of compound, tissue, fish size and species, environmental conditions and production system [44].

Severe thermal processing and cooking procedures (boiling, frying, steaming) increase the oxidation of carotenoids, PUFA and other lipid bioactives in fish, causing a reduction of its nutritional quality [38,39].

### 3.4. Blueberries

The main nutrients that blueberries (*Vaccinium* Section *Cyanococcus*) deliver, according to websites, are fiber, vitamin C and minerals in general; vitamins E and K, manganese and potassium were mentioned fewer times (Table 10 and Table 11). These components have also been reported in scientific publications, together with others not cited by sites such as vitamins of B complex, E and A, selenium, zinc and iron [45,46].

The effects on health and in disease prevention of blueberries indicated in selected websites are shown in Table 12; they highlight their antioxidant activity and their positive impact on cardiovascular diseases prevention, cancer risk reduction and weight loss.

The health-promoting potential of blueberries intake is mainly related to their high content in antioxidant compounds. They are a good source of phenolic compounds, primarily the flavonoids anthocyanins, which have been most commonly associated with their health effects. In addition, several types of other flavonoids (catechin, quercetin, kaempferol, etc.), phenolic and cinnamic acids, and proanthocyanins have been reported [45,47]. The antioxidant capacity of blueberries compounds has been linked to anticancer and anti-mutagenic effects due to how they provide certain protection against DNA oxidation and tumor cell proliferation, with a significant inhibitory effect for some types of cancer. However, the studies have been carried out using blueberries extracts in tumor cell lines and laboratory animals; *in vivo* investigation on humans and with whole fruits intake are necessary to confirm these effects. Besides, longer continuous ingestion was necessary to obtain positive results [45,48,49].

Flavonoids ingestion has been associated with cardiovascular diseases protection by the improvement of blood flow and endothelial function, and platelet aggregation inhibition. The effects of blueberry intake seem to be affected by the duration of the study. Anthocyanins have been linked with a positive effect on vascular function, hyperlipidemia and a lower risk of myocardial infarction after blueberries intake in dried form, as drink or extracts. However, the effect of blueberries consumption on the reduction of blood pressure is not clear [48,50,51].

Neuroprotective properties of blueberries (improvement of memory and learning skills, and prevention of neurodegenerative diseases) were mentioned by nine sites (Table 12). Memory enhancement (probably due to the increase in synaptic plasticity), favorable effects on cognitive function and improved motility in older adults have been reported. This effect, together with improved learning, has also been described in laboratory animals. Again, anthocyanins seem to be responsible for it, acting as antioxidants and anti-inflammatory compounds [45,46,52].

In relation to weight loss, blueberries consumption appears to have no impact on weight gain or fat accumulation in laboratory animals; by contrast, anthocyanin extracts may reduce both parameters [45].

Other beneficial effects, mentioned fewer than five times on sites, are also reflected in the scientific literature. These are anti-inflammatory and antimicrobial activity, digestive health and immunity improvement, diabetes risk reduction and anti-aging effects [45,47,48,53]. No information about positive effects on skin and eye health in the literature was found.

The results regarding some health benefits of blueberries consumption are variable and seem to be affected by the trial period and characteristics of participants (sex, age, overall health). Besides, the dose of anthocyanins and the matrix in which they are contained (powder, fresh fruit, drink) could influence the rate of anthocyanins absorption. In general, scientific studies conclude that frequent consumption of this fruit has positive effects on human health, but further investigation is needed [46,52].

### 3.5. Avocado

According to websites, the most highlighted nutrients of avocado (*Persea americana* Mill.) are monounsaturated fatty acids (MUFA) or ‘healthy fats’ (mentioned 12 and 8 times, respectively), followed by fiber, magnesium and potassium (7 and 6 times) (Table 10 and Table 11). The vitamin E, K, C and B group, copper and manganese are cited fewer than five times. Avocado is cited in the scientific literature as a good source of these nutrients [54,55]. Four sites named the avocado as a good source of PUFA; however, this is not confirmed by scientific information [56]. Plant variety, environmental conditions, maturity stage, soil composition and fertilization are important factors that influence the composition of avocado [57].

High MUFA content also appears to improve the absorption of other lipophilic compounds (phytosterols, vitamin E) of avocado or those that have been consumed together with it (provitamin A), which could reinforce the beneficial health effects of this fruit [56].

The main effects on health of avocado, in accordance with the selected websites, are cardiovascular diseases prevention (14 mentions) and diabetes risk reduction (5 mentions); besides, antioxidant activity, cancer risk reduction and eye health promotion were cited four times (Table 12).

Promotion of vascular health has been attributed to carotenoids, tocopherol, phenolic compounds and phytosterols contents of avocado. Xanthophylls seem to have a beneficial effect by suppressing blood vessel damage due to how they diminish the quantity of oxidized low-density lipoproteins. Phytosterols, mainly β-sitosterol in avocado, have a cardioprotective effect by inhibiting cholesterol absorption. Phenolic compounds also contribute to cardiovascular disease prevention by reducing platelet aggregation. In clinical studies, avocado consumption has shown positive effects due to the decrease in triglycerides and serum cholesterol levels, and the reduction of weight gain in healthy and hypercholesterolemic human subjects. The high content of the oleic acid of this fruit has also been related to its cardioprotective effect [54,56]. However, clinical studies reported heterogeneous results in some cardiovascular health indicators, which can be ascribed to differences in the amount of avocado consumed, the limited number of participants and the lack of long-term cardiovascular disease evaluations [55]. Similar observations have been reported regarding the effect of avocado intake on weight loss and on metabolic parameters related to obesity [58].

The type 2 diabetes risk-reduction effect of avocado intake has been attributed to its high content in indigestible carbohydrates; its inclusion in the diet of laboratory animals decreases the blood sugar levels [54] and improves the glycemic profiles in humans [56].

Regarding the other health benefits, avocado seems to have *in vitro* cytotoxic properties against several cancer cells (breast, colon, liver, esophageal, ovarian, prostate, etc.) and anticancer effects have also been reported in laboratory animals. However, these investigations have been carried out using extracts, many times from non-edible parts of the fruit. The studies suggest the potential of avocado extracts in cancer treatment, but clinical research about the effects of its intake in cancer prevention is needed [54]. Carotenoids lutein and zeaxanthin are able to slow down several eye diseases’ progress related to aging (cataracts, macular degeneration) [56].

### 3.6. Chia

The nutrients of chia (*Salvia hispanica* L.) seeds highlighted on selected sites are protein and healthy fats, in particular, linolenic acid, fiber and minerals, especially magnesium (Table 10 and Table 11). According to scientific information, chia seeds contain large amounts of dietary fiber and lipids, which are rich in polyunsaturated fatty acids, mainly α-linolenic acid. They also have significantly high protein content compared to other seeds; this protein possesses a good digestibility. They are considered a rich source of calcium and also contain a significant amount of macro- and microelements, and vitamins (A, E, C and B complex); however, the number of mentions of these nutrients in sites was lower than 3 [59,60].

As occurs with other plant-based foods, the contents of nutrients vary depending on several factors, such as plant origin, environmental conditions, soil characteristics, harvesting time and storage and drying of seeds [59]. Roasting of chia seeds affects negatively the bioactive properties of chia lipids (oil), decreasing PUFA and phenolic compounds contents [61].

The main beneficial effects of chia seeds consumption cited by websites are antioxidant activity and cardiovascular disease protection (8 and 9 times, respectively) (Table 12). Other actions, mentioned 3 times, are weight control, presence of anti-inflammatory compounds, diabetes risk reduction and bone-health maintenance. Most of these actions are referred to in the scientific literature, both in animal models and clinical studies: antiobesity, antioxidant, anticarcinogenic and hypotensive effects, cardiovascular protection and improvement of lipid and glucose [59,60]. Weight control has been attributed in part to the hydration properties of the mucilage that the seeds release after soaking in water [62], when they are consumed in this form. The mucilage increases satiety probably owing to the delay in gastric emptying [63]. The antioxidant activity of chia seeds is attributed to their content in phenolic compounds (phenolic acids, flavonoids, catechin derivatives) and carotenoids, and also, in antioxidant proteins and peptides. These compounds could protect against heart disease and cancer, among other pathologies [64]. Consumption of foods with high α-linolenic acid content, such as chia, shows beneficial effects on decreasing atherogenic lipids and lipoproteins, blood pressure and inflammation markers; these benefits seem related to the conversion of α-linolenic acid to long chain n-3 PUFAs (EPA and DHA) and also, to its intrinsic effects (cardiac ion channels modulations, conversion into bioactive oxylipins) [65].

There is some controversy about the health benefits of chia seeds consumption in *in vivo* studies [66]. In unbalanced diet animal studies, the use of chia as a source of α-linolenic acid causes fat redistribution and a decrease in the abdominal area, reducing cardiovascular disease risk. Seeds consumption also increases total antioxidant capacity. Besides, chia intake improves glucose tolerance and insulin sensitivity, having an impact on lipogenesis. The bioactive compounds and their interactions involved in these effects are still unknown [61]. Chia also shows hypolipidemic effects [67]. In a meta-analysis of clinical studies [68], it is reported that the doses and form of chia seeds and the selection of the participants may influence the results. The improvement of lipid parameters is higher in healthy subjects than in obese/overweight ones. Chia consumed in ground form seems to be more beneficial for blood pressure and glucose, and lipid parameters morethan whole seed. These effects are more significant when chia is taken in high doses. In both animal and human clinical studies, correction of problems in the design and methodology of the trials must be carried out in order to establish clearly the health effects of chia consumption [61,68].

### 3.7. Walnuts

The most important nutrients of walnuts (*Juglans regia* L.) cited by sites are protein (mentioned 9 times), health fats (10 times) especially linolenic acid (7 times), and fiber (11 times) (Table 10). Walnuts are a good source of these compounds according to scientific literature [69]. Moreover, walnuts constitute a source of fiber, iron, zinc, potassium and vitamins E, B_3_ and B_5_; besides, they are high in vitamins B_1_, B_6_, biotin and folate, and in magnesium, manganese, copper and phosphorus [70]. However, only a few (1 to 4) sites consider walnuts an important resource for these nutrients.

According to selected websites, the main effects on health promotion of walnuts are the antioxidant activity and the prevention of cardiovascular diseases; they were mentioned 11 times each (Table 12). Other beneficial effects are the anti-inflammatory activity and the improving of memory and learning skills (cited 3 and 4 times, respectively). All these properties have been related to the high content in bioactive compounds, such as phenolic compounds (quercetin, ellagic acid, ellagitannins, cyanidin and proanthocyanidins), phytosterols, γ-tocopherol, dietary fiber, protein (high L-arginine content), phytomelatonin and α-linolenic fatty acid [69,71]. Walnuts consumption has been associated with significant decreases in triglyceride, total cholesterol and LDL cholesterol contents in middle-aged and older adults [72]; this improvement in lipid profiles is associated with the reduction of cardiovascular diseases risk [70,73].

Some compounds in walnuts have health-promoting effects, although these properties of the isolated components are not always translated into health enhancement or disease risk reduction. PUFA boost brain function and cognitive function improvement has been observed in humans, mainly in long-term walnut consumption [71,74]. Ellagitannins release ellagic acid, which shows antioxidant properties and thus provides anti-inflammatory protection; after, it is metabolized by intestinal bacteria to urolithins, which also have antioxidant and anti-inflammatory properties [69]. However, walnuts consumption appears not to be associated with inflammatory markers and glucose homeostasis in clinical studies [72], albeit their inclusion in diets produces modifications in human gastrointestinal microbiota and reduces proinflammatory factors derived from the microbial activity [74].

Disease prevention and health promotion of walnuts intake in clinical trials seem to be prone to some bias, due to the wide dosage range, differences in diets among participants from different geographical regions and other defects in studies’ design and methodology. For these reasons, including walnuts in one’s diet probably has positive effects on health, although more investigations are needed [72,73].

### 3.8. Beans

The main nutrients provided by beans (*Phaseolus vulgaris* L.), in accordance with websites, are protein (cited 16 times), fiber (17 times), vitamins (in general, 5 times) and minerals (in general, 5 times), particularly iron (5 times) and magnesium (6 times) (Table 10 and Table 11). Beans contain high amounts of protein, comparable, in quantitative terms, to meat, although the content of some essential amino acids (methionine and tryptophan) is not very elevated. The digestibility of their protein is impaired by protease inhibitors and tannins, but an adequate cooking increases its bioavailability [75].

Beans are a very good source of folate, but only 4 sites mentioned this vitamin. Regarding minerals, they contain amounts of iron, magnesium and potassium, as is reflected in 4 to 6 sites, but also of calcium, phosphorus, copper, manganese, selenium and zinc [75], which are not mentioned at all.

Phytochemicals content, and as a result, the potential effects of beans consumption on health, is affected by genetic factors (higher content in pigmented coat varieties), environmental growing conditions, storage, processing methods and the specific bioaccessibility and bioavailability of each component. Fermentation, germination, extrusion and roasting increase polyphenol content [76,77,78].

There is no information on the selected websites about beans phytochemical compounds that may interfere in the bioavailability of some nutrients or may produce health problems under specific conditions. As stated above, beans contain trypsin inhibitors, but also the α-amylase inhibitor, which decrease the digestibility of starch, and phytic acid and oxalate, which disfavor the absorption of minerals. Besides, beans also contain lectins, which can cause health problems [75]. Fortunately, most antinutritional factors can be reduced significatively by thermal processing, storage, milling, fermentation and germination [78]. An adequate cooking process includes soaking (discarding the soaking water) and a sufficiently long cooking at high temperature [79].

Beans have two main positive effects on health with regard to cardiovascular diseases protection and type 2 diabetes risk reduction (cited 8 and 5 times, respectively) according to websites information (Table 12). Secondarily, weight control and cancer risk reduction are also mentioned (4 and 3 times, respectively).

Beans are a rich source of bioactive components such as carbohydrates (polysaccharides and oligosaccharides), proteins, phenolic compounds (phenolic acids, flavonoids and proanthocyanidins) and various minerals and vitamins that have the potential to improve human health [75,76].

With respect to cardiovascular protection, common beans reduce LDL cholesterol values and the risk of disease in variable percentage of reduction depending on the studies [80]. Several studies have demonstrated that bean intake decreases postprandial glucose levels, but frequently, the results are not significative. Benefits related to type 2 diabetes amelioration have been associated with the high insoluble fiber content of beans and some minerals, mainly iron and zinc [80]. The high quantity of resistant starch of beans may enhance glycemic and blood pressure control and helps to maintain healthy gut microbiota. Short-chain fatty acids, products of intestinal bacteria metabolism, seem to be involved in the improvement of glucose tolerance and have anti-obesity effects [75,81]. Beans peptides, released by gastrointestinal proteases during digestion, may also provide antithrombotic and antihypertensive effects and prevent hyperglycemia [81]. At least at present, there is no clear evidence that beans consumption reduces the probability of developing cancer, including colorectal cancer [80].

As has been observed in meta-analysis of other food products, the clinical studies showed differences in bean variety, growing conditions, the characteristics and number of participants evaluated, and the form of administration (the whole seed or as an extract). These aspects, together with the lack of measurements standardization, make it difficult to determine the health effects of bean consumption [80].

### 3.9. Fermented Milks

Information in selected sites highlights the protein and calcium contents of fermented milks (Table 10 and Table 11). They are a good source of these nutrients; protein content in yogurt is higher than that of milk due to the addition of milk protein or skimmed milk powder. Moreover, milk proteins are a great source of essential amino acids. The acidic pH value of yogurt increases calcium absorption and its bioavailability. Fermented milks are also a source of vitamins (D, A, E, B_2_, B_3_, B_12_ and folates), and minerals other than calcium (phosphorus, zinc, magnesium, potassium) [82]. Most of these nutrients are not cited by websites.

Digestive health and immunity improvement are the leading health effects of fermented milks (mainly, yogurt and kefir) according to selected websites, with 12 and 8 mentions, respectively (Table 12).

Traditional yogurt, the product of fermenting milk with symbiotic bacteria *Lactobacillus delbrueckii* subsp. *bulgaricus* and *Streptococcus thermophilus*, is considered probiotic because its starter cultures are able to survive in the gastrointestinal tract and exhibit health benefits. However, not all yogurt cultures possess the same characteristics, and their growing is also influenced by the temperature and fermentation period, which has consequences in sensory properties of the product. Yogurt has also been used as a carrier of other probiotic cultures, by total or partial substitution on the traditional cultures [83]. Kefir natural microbiota are lactic acid bacteria, yeasts and *Acetobacter*, the most abundant being the microorganisms of the genus *Lactobacillus*. The composition of this microbiota is different depending on the geographical region, but the interactions among kefir microorganisms from different locations and their functions are very similar [84].

Regarding digestive disorders amelioration and digestive health, human clinical studies refer to significant findings in diarrhea and inflammatory bowel disease (by gut microbiota modulation), gastroesophageal reflux disorder and peptic ulcer disease (through eradication of the infection by *Helicobacter pylori*) after the consumption of yogurt with prebiotics, kefir and fermented milks with diverse probiotics [85]. Gut microbiota appears to be modified preferentially in subjects with gut health alterations [86].

Fermented milks enhance immunity response against viruses and pathogen bacteria in humans; they also seem to reduce some allergic disorders by gut microbiome modulation, but this requires more investigation [85]. It is believed that kefir intake and the metabolites of its microbiota (short-chain fatty acids, extracellular polysaccharide kefiran, polypeptides, lactic acid) may regulate the physiological functions of the gastrointestinal immune system and can promote secretory immunoglobulin A (the main antibody of intestinal mucosa immunity) production. Nevertheless, this effect on immunity has not been able to be demonstrated through experimental studies using laboratory animals fed with Kefir. Besides, the gut colonization capability of Kefir microbiota has not been determined [84].

Other health benefits of fermented milks intake have been reported in the literature; however, they are hardly ever mentioned in sites. These are cardioprotective properties (reduction of LDL cholesterol and blood pressure), anti-obesity and anti-diabetes effects, and bone health and cognitive improvement [85].

Health-promoting effects of fermented milks may be partly caused by the microbial synthesis and release of bioactive compounds, such as peptides, bacteriocins, exopolysaccharides, conjugated linoleic acid and B complex vitamins (folate, B_2_, B_12_). Bioactive peptides have shown antimicrobial, antioxidative and immune-modulatory properties [87]. Regular yogurt intake has been related to lower cardiovascular disease risk and linked with peptides content; these peptides may have originated during fermentation or digestion of yogurt. Bioactive peptides have also been detected in kefir. Cardioprotective effects of fermented milk peptides have been mainly studied in laboratory animals, with the oral administration of isolated compounds [88]. Kefir has a wide range of probiotic health effects based on the metabolites produced by microbial cultures, which includes hypoglycemic, anticancer, anti-inflammatory and antibacterial activities [84]. Milk calcium improves the HDL:LDL cholesterol ratio, and caseins and whey proteins could also regulate blood pressure. Yogurt consumption is associated with a type 2 diabetes risk decrease due to its content of vitamin K_2_ [89].

Some human health benefits of fermented milks consumption have been experimentally established, but, in other cases, a disparity between the results of animal and human trials has been observed. This discrepancy is probably due to the variety of diets and lifestyles of human participants, the differences in gut microbiome and the type of controls (milk or nonmilk) used in the studies [85,87].

### 3.10. Garlic

Regarding the nutritional composition of garlic (*Allium sativum* L.), none of the nutrients was mentioned more than 3 times by selected sites (Table 10 and Table 11). All of them are listed in the scientific literature. Garlic contains fiber, minerals (zinc, phosphorus, potassium, calcium, iron, selenium, magnesium and manganese) and vitamins (A, C and B complex) [90].

Garlic, according to the information provided by websites, gives cardiovascular diseases protection, has antimicrobial properties, anti-cancer activity, and improves immunity (cited 11, 10, 8 and 8 times, respectively) (Table 12).

Garlic contains a wide variety of bioactive chemicals, such as organosulfur compounds, phenolic compounds (polyphenols, flavonoids, flavanols, tannins), saponins and polysaccharides [90], which are responsible for its beneficial effects on health. Nevertheless, bioactive compounds composition and concentration change depending on the production process applied to raw cloves to obtain derivatives and extracts. Many factors affect the bioactivity of these compounds: preparation, extraction conditions, temperature or storage [91]. Organosulfur compounds are responsible for many of the health properties attributed to garlic and are strongly influenced by processing. The non-volatile organosulfur compound alliin is only detected in undamaged bulbs and is transformed into other chemicals by maceration or crushing, for example, in allicin, a volatile organosulfur, which decomposes in various diallyl sulfides. Due to the chemical reactions of these compounds, garlic extracts show higher biological properties than other derivatives. Whole raw garlic bulbs are also transformed into black garlic by heating at a high temperature under controlled humidity conditions and for a period of time longer than one month. In the course of the treatment, several fresh garlic components are converted by means of the Maillard reaction into more bioactive compounds. Due to this treatment, the polysaccharide is degraded, while S-allyl cysteine, total polyphenols and flavonoids contents increase compared with the raw material, among other changes [91,92,93].

Garlic has antibacterial, antifungal and antiviral activities, probably due to its organosulfur compounds. Allicin administration reduces the severity and duration of respiratory infections in both adults and children [92]. Garlic may promote cardiovascular health by the improvement of lipid profile (reduction of triglycerides and cholesterol levels, increase of HDL values), blood pressure and endothelial function. Regular raw garlic consumption may decrease pre-hypertension risk. Again, allicin has an important role by means of platelet aggregation inhibition and antihypertensive effect. The intensity of the antiplatelet effect can depend on the garlic-derived product used in trials, being negligible in some clinical studies that used garlic oil or tablets. Antioxidant activity of phenolic and organosulfur compounds of garlic and the prebiotic effect of garlic extracts on gut microbiota may also participate in these benefits [90,92,94].

With respect to cancer risk reduction, garlic seems to show effectivity against breast, ovarian, renal, liver, esophageal, colorectal and gastric cancers and produces symptomatic relief. Organosulfur compounds have been associated with this effect. The mechanisms comprise inhibition of angiogenesis, cell growth and migration, regulation of carcinogen metabolism and induction to apoptosis [91,92]. However, more clinical studies are needed in order to draw firm conclusions about the effects of garlic intake on cancer [95].

Polysaccharides seem to be the principal garlic compounds with immunomodulatory effects, although allicin also appears to be important. Polysaccharides activity is higher in fresh garlic than in black garlic and depends on the dose of garlic and the type of derivatives administered. Enhancing of immune function by allicin has been reported as a factor for the elimination of cancer metastasis. Besides, the protective effect of garlic compounds against infections has also been related with their capability of activating the immune system [91,92,96].

It is important to note that the experimental research using laboratory animals and the clinical studies about the effect of garlic on health frequently use garlic extracts or some purified bioactive compounds in the trials, and rarely unprocessed garlic. It is difficult to extrapolate the results to a normal form of garlic intake as meal seasoning or as raw clove by consumers.

## 4. Conclusions

In general terms, the information provided by websites about the health benefits associated with the consumption of most cited ‘superfoods’ is quite correct according to scientific literature, but with nuances. These beneficial actions are based, on many occasions, with those of bioactive compounds contained in foods that have been checked *in vitro* and in experiments using laboratory animals. The clinical studies evaluating the health effects of pure bioactive compounds or extracts are limited, and even more, those in which direct ‘superfood’ intake has been assessed. Besides, human trials vary in design, methodology, participants characteristics (diet, lifestyle), the form and serving size of the food administered, duration and markers measured, among others. All these factors make it difficult to draw conclusions about the effects of ‘superfoods’ on health.

Moreover, many factors can influence the presence and concentration of bioactive components in these ‘superfoods’. In plant-based foods, they are related with growing conditions, storage and varieties; in animal foods, the variations may stem from feeding or way of living (e.g., farmed or wild). Bioactive chemicals are also affected by processing and cooking methods, and their bioavailability depends on interactions among them and with other food compounds.

It may be concluded that the information provided by websites is presented in a very simplified form, although in general, it is not incorrect. Information needs to be easy to understand by consumers but, at the same time, it should avoid creating false expectations about health improvements. In any case, the consumption of these foods as a part of a balanced and varied diet can be beneficial to human health.

## Figures and Tables

**Table 1:** Classification of the groups of superfoods by the number of times that were mentioned in selected web pages.

Group of Superfoods	Number of Times Mentioned				
	First Page	Second Page	Any Time	Past Year	Total Mentions
Leafy greens and cruciferous vegetables	122	32	108	46	154
Whole grain cereals, seeds and cereals	71	33	67	37	104
Berries	65	25	65	25	90
Fish and seafood	44	17	37	24	61
Other fruits	41	16	33	24	57
Nuts	46	6	34	18	52
Legumes	33	14	25	22	47
Spices and herbs	22	10	20	12	32
Fermented foods	23	6	14	15	29
Teas and infusions	13	5	9	9	18
Fats and oils	12	4	7	9	16
Other vegetables and plant-based foods ^1^	87	23	59	51	110
Other animal-based foods and other ^2^	14	7	12	9	21

^1^ Miscellaneous group of 19 plant-based foods. ^2^ Miscellaneous group of 7 animal-based foods.

**Table 2:** Classification of cruciferous vegetables and leafy greens by the number of times that were mentioned on selected web pages.

Food	Number of Times Mentioned				
	First Page	Second Page	Any time	Past Year	Total Mentions
**Cruciferous vegetables**					
Kale	21	6	19	8	27
Broccoli	15	6	13	8	21
Collard greens	11	4	9	6	15
Cauliflower	6	3	6	3	9
Arugula	6	1	5	2	7
Brussel sprouts	5	2	3	4	7
Mustard greens	5	1	6	0	6
Cabbage	6	0	4	2	6
Watercress	4	1	3	2	5
Turnip greens	3	0	2	1	3
Bok choy	2	0	2	0	2
Rutabaga	1	0	1	0	1
**Leafy greens**					
Spinach	20	5	18	7	25
Swiss chard	12	3	12	3	15
Beet greens	2	0	2	0	2
Dandelion greens	2	0	2	0	2
Lettuce	1	0	1	0	1

**Table 3:** Classification of fish and seafood by the number of times that were mentioned on selected web pages.

Fish and Seafood	Number of Times Mentioned				
	First Page	Second Page	Any Time	Past Year	Total Mentions
Salmon	17	7	15	9	24
Sardines	6	3	6	3	9
Mackerel	4	1	3	2	5
Herring	4	1	4	1	5
Tuna	3	1	2	2	4
Trout	4	0	3	1	4
Anchovies	1	1	1	1	2
Oysters	1	1	1	1	2
Cod	2	0	1	1	2
Shellfish	1	0	0	1	1
Shrimp	1	0	0	1	1
Halibut	0	1	1	0	1
Salmon roe	0	1	0	1	1

**Table 4:** Classification of fruits by the number of times that were mentioned on selected web pages.

Fruit	Number of Times Mentioned				
	First Page	Second Page	Any Time	Past Year	Total Mentions
**Berries**					
Blueberries	16	7	18	5	23
Cranberries	10	3	9	4	13
Raspberries	8	2	7	3	10
Acai berries	6	3	7	2	9
Strawberries	6	3	6	3	9
Blackberries	6	2	6	2	8
Goji berries	4	4	6	2	8
Elderberries	3	1	1	3	4
Tart cherries	2	0	2	0	2
Aronia	2	0	1	1	2
Lingonberries	1	0	1	0	1
Maqui berries	1	0	1	0	1
**Other fruits**					
Avocado	14	7	12	9	21
Citrus fruits	5	2	2	5	7
Apples	5	2	5	2	7
Pomegranate	3	2	3	2	5
Watermelon	4	1	3	2	5
Bananas	2	1	1	2	3
Coconut	2	1	2	1	3
Papaya	2	0	1	1	2
Cherries	1	0	1	0	1
Prunes	1	0	1	0	1
Figs	1	0	1	0	1
Pineapple	1	0	1	0	1

**Table 5:** Classification of whole grain cereals, seeds and pseudocereals by the number of times that were mentioned on selected web pages.

Whole Grain Cereals, Seeds and Pseudocereals	Number of Times Mentioned				
	First Page	Second Page	Any Time	Past Year	Total Mentions
Chia	11	6	10	7	17
Oats	11	5	9	7	16
Quinoa	9	6	10	5	15
Flaxseeds	8	5	8	5	13
Hemp seeds	5	2	5	2	7
Brown rice	4	1	3	2	5
Sunflower seeds	4	0	2	2	4
Buckwheat	3	1	4	0	4
Barley	3	1	4	0	4
Farro	2	1	2	1	3
Pumpkin seeds	2	1	1	2	3
Teff	1	2	1	2	3
Amaranth	1	1	1	1	2
Bulgur wheat	2	0	2	0	2
Whole wheat	2	0	1	1	2
Spelt	1	0	1	0	1
Sesame seeds	1	0	1	0	1
Grape seeds	1	0	1	0	1
Sorghum	0	1	1	0	1

**Table 6:** Classification of nuts, legumes and fermented foods by the number of times that were mentioned on selected web pages.

Food	Number of Times Mentioned				
	First Page	Second Page	Any Time	Past Year	Total Mentions
**Nuts**					
Walnuts	12	3	8	7	15
Almonds	12	1	9	4	13
Brazil nuts	7	0	5	2	7
Pistachios	2	2	4	0	4
Cashews	4	0	3	1	4
Pecans	3	0	2	1	3
Macadamia nuts	3	0	1	2	3
Peanuts	3	0	2	1	3
**Legumes**					
Beans	12	4	8	8	16
Lentils	10	4	6	8	14
Peas	5	2	4	3	7
Chickpea	3	3	4	2	6
Soybeans	3	1	3	1	4
**Fermented food**					
Fermented milks (yogurt, kefir)	14	4	8	10	18
Kombucha and kimchi	2	1	2	1	3
Sourdough + other breads	2	0	0	2	2
Sauerkraut	1	0	1	0	1
Vinegar	1	0	0	1	1
Olives	1	0	1	0	1

**Table 7:** Classification of spices and herbs, teas and infusions, and fats and oils by the number of times that were mentioned on selected web pages.

Food	Number of Times Mentioned				
	First Page	Second Page	Any Time	Past Year	Total Mentions
**Spices and herbs**					
Turmeric, curcumin	8	4	5	7	12
Ginger	7	3	7	3	10
Cinnamon	3	1	3	1	4
Black pepper	1	1	1	1	2
Peppermint	1	0	1	0	1
Parsley	1	0	1	0	1
Oregano	1	0	1	0	1
Tarragon	0	1	1	0	1
**Teas and infusions**					
Green tea	9	3	7	5	12
Matcha	3	2	2	3	5
Coffee	1	0	0	1	1
**Fats and oils**					
Olive oil	6	2	4	4	8
Coconut oil	3	1	1	3	4
Other fats (grass-fed butter, ghee, flaxseed oil, fish oil)	3	1	2	2	4

**Table 8:** Classification of other plant-based foods by the number of times that were mentioned on selected web pages.

Food	Number of Times Mentioned				
	First Page	Second Page	Any Time	Past Year	Total Mentions
Garlic	14	2	9	7	16
Seaweed	10	4	10	4	14
Mushrooms	11	2	4	9	13
Sweet potato	8	2	6	4	10
Dark chocolate	6	4	7	3	10
Tomato	7	2	5	4	9
Beet	5	2	3	4	7
Pumpkin	5	1	5	1	6
Peppers	3	1	2	2	4
Carrots	3	0	1	2	3
Artichoke	2	0	2	0	2
Maca	1	1	0	2	2
Onions	2	0	0	2	2
Wheat grass	1	1	2	0	2
Potato	1	0	0	1	1
Eggplant	0	1	0	1	1
Mankai	1	0	0	1	1
Asparagus	1	0	0	1	1
Cucumber	1	0	0	1	1

**Table 9:** Classification of other animal-based foods and other foods by the number of times that were mentioned on selected web pages.

Food	Number of Times Mentioned				
	First Page	Second Page	Any Time	Past Year	Total Mentions
Eggs	9	3	7	5	12
Water	3	0	2	1	3
Raw milk	1	1	1	1	2
Bone broth	0	1	1	0	1
Nutritional yeast	0	1	1	0	1
Liver	0	1	0	1	1
Meat	1	0	0	1	1

**Table 10:** Macronutrients of ‘superfoods’ and number of selected websites that mentioned each one.

Macronutrient	Food									
	Kale	Spinach	Salmon	Blueberries	Avocado	Chia	Walnuts	Beans	Fermented Milks	Garlic
Protein	1	1	10	-	-	10	9	16	11	-
Amino acids	-	-	-	-	-	-	1	-	-	-
Fats (healthy)	-	-	2	-	8	6	10	1	-	-
Monounsaturated fatty acids	-	-	-	-	12	-	1	-	-	-
Polyunsaturated fatty acids	-	-	-	1	4	1	-	-	-	-
Linoleic acid	-	-	-	-	-	-	-	-	-	-
Linolenic acid	-	1	-	-	-	10	7	-	-	-
EPA/DHA	-	-	25	-	-	-	-	-	-	-
Carbohydrates	-	-	-	-	-	-	-	-	-	-
Fiber	11	12	-	7	7	14	11	17	-	1

**Table 11:** Micronutrients of ‘superfoods’ and number of selected websites that mentioned each one.

Micronutrient	Food									
	Kale	Spinach	Salmon	Blueberries	Avocado	Chia	Walnuts	Beans	Fermented Milks	Garlic
Vitamins (in general)	2	2	5	4	3	4	3	5	-	-
Vitamin A/provitamin A	10	9	1	-	-	-	-	-	-	-
Vitamin D	-	2	3	-	-	-	-	-	2	-
Vitamin E	2	2	-	1	2	1	3	-	-	-
Vitamin K	6	5	-	2	2	-	-	-	1	-
Vitamins B	-	-	-	-	-	-	1	2	2	-
Vitamin B1 (thiamine)	-	-	-	-	-	1	1	-	-	-
Vitamin B2 (riboflavin)	-	-	-	-	2	-	1	-	-	-
Vitamin B3 (niacin)	-	-	-	-	2	1	-	-	-	-
Vitamin B5 (pantothenic acid)	-	-	-	-	1	-	-	-	-	-
Vitamin B6 (pyridoxine)	-	1	-	-	1	-	1	-	-	2
Vitamin B12 (cyanocobalamin)	-	-	2	-	-	-	-	-	-	-
Biotin	-	-	-	-	-	-	-	-	-	-
Folic acid /folate	4	6	-	-	3	-	1	4	1	-
Vitamin C	12	9	-	6	3	1	-	-	-	3
Minerals (in general)	1	2	1	3	3	6	4	5	-	-
Calcium	10	6	-	-	-	1	1	1	10	-
Iron	5	6	-	-	-	2	1	5	-	-
Phosphorus	-	-	-	-	-	2	1	-	2	-
Iodine	-	-	1	-	-	-	-	-	-	-
Magnesium	3	4	-	-	6	5	3	6	1	-
Zinc	2	3	-	-	-	2	1	2	-	1
Selenium	-	-	4	-	-	-	1	1	-	3
Copper	-	-	-	-	1	-	1	1	-	-
Manganese	1	-	-	2	1	1	2	2	-	2
Potassium	2	3	2	1	6	-	1	4	2	-

**Table 12:** Effects on health and disease prevention of ‘superfoods’ and number of selected web sites that mentioned each one.

Effect	Food									
	Kale	Spinach	Salmon	Blueberries	Avocado	Chia	Walnuts	Beans	Fermented Milks	Garlic
Weight loss/control	-	1	-	5	3	3	1	4	-	1
Antioxidant activity	7	6	1	18	4	8	11	2	-	1
Antimicrobial activity	1	1	-	2	-	-	-	-	-	10
Cardioprotective effect/cardiovascular diseases protection	8	10	16	14	14	9	11	8	2	11
Cancer risk reduction	9	9	3	8	4	2	2	3	1	8
Anti-inflammatory activity	1	3	7	4	1	3	3	1	3	3
Digestive disorders/digestive health	5	3	-	2	1	2	-	-	12	1
Immune-related disorders/immunity improving	2	2	2	4	1	-	-	1	8	8
Improving memory and learning skills	-	1	7	4	2	1	4	-	-	-
Prevention of neurological or neurodegenerative diseases	1	-	2	5	-	-	1	1	-	1
Antidepressant action	1	-	2	-	-	-	-	-	-	-
Anti-aging	-	-	-	2	-	1	-	-	-	-
Type 2 diabetes risk reduction	3	1	1	4	5	3	2	5	-	2
Prevention of endocrinological and metabolic disorders	1	-	-	-	-	-	-	1	-	-
Effect on urinary and genital system	-	-	-	-	-	-	-	-	-	-
Bone health/osteoporosis	2	5	1	-	1	3	-	-	1	-
Skin health	-	1	3	1	3	1	2	1	-	2
Eyes health	1	3	1	3	4	-	-	1	-	1

## Data Availability

Data is contained within the article or supplementary materials.

## Supplementary Materials

The following supporting information can be downloaded at: https://www.mdpi.com/article/10.3390/foods12030546/s1, Table S1: List of selected web pages found in the first page of search engines; Table S2: List of selected web pages found in the second page of search engines.
