# Supplementary material for: ‘Superfoods’: Reliability of the Information for Consumers Available on the Web
Source: Foods. 2023 Jan 26;12(3):546. doi: 10.3390/foods12030546 (PMC9914617; doi:10.3390/foods12030546)
Supplement: Supplementary file 1 [file foods-12-00546-s001.zip › foods-2144219-supplementary.docx]

**Table S1.** List of selected web pages found in the first page of search engines.

| **Order No.** | **Search engine** | **Year** | **URL** |
| --- | --- | --- | --- |
| 1 | Google | 2018 | https://www.health.harvard.edu/blog/10-superfoods-to-boost-a-healthy-diet-2018082914463 |
| 2 | Google | 2018 | https://www.healthline.com/nutrition/true-superfoods |
| 3 | Google | 2019 | https://www.medicalnewstoday.com/articles/303079 |
| 4 | Google | 2020 | https://www.everydayhealth.com/photogallery/superfoods.aspx |
| 5 | Google | 2020 | https://www.womansday.com/food-recipes/food-drinks/g2211/best-superfoods/ |
| 6 | Google | 2021 | https://health.clevelandclinic.org/what-is-a-superfood/ |
| 7 | Google | 2019 | https://www.livescience.com/34693-superfoods.html |
| 8 | Google | 2022 | https://en.wikipedia.org/wiki/Superfood |
| 9 | Google | 2022 | https://greatist.com/health/25-greatist-superfoods-and-why-theyre-super |
| 10 | Google ^1^ | 2022 | https://www.gousto.co.uk/blog/top-10-superfoods |
| 11 | Google ^1^ | 2021 | https://www.aarp.org/health/healthy-living/info-2021/superfoods-for-adult-health.html |
| 12 | Google ^1^ | 2021 | https://www.milliondollarvegan.com/top-10-healthiest-foods/ |
| 13 | Google ^1^ | 2012 | https://www.andreas-skincare-bodytherapy.com/blog/the-13-most-powerful-superfoods |
| 14 | Google ^1^ | 2012 | https://www.huffpost.com/entry/superfoods-healthy-food_n_1998293 |
| 15 | Google | 2022 | https://www.aarp.org/health/healthy-living/info-2022/superfoods-for-increased-energy.html |
| 16 | Google | 2021 | https://www.medicinenet.com/the_top_12_superfoods_what_are/article.htm |
| 17 | Bing | 2020 | https://www.healwithfood.org/superfoods/ |
| 18 | Bing | 2018 | https://superfooddrinks.org/superfoods-list/ |
| 19 | Bing | 2016 | https://www.avogel.co.uk/food/what-are-superfoods-the-ultimate-guide |
| 20 | Bing | 2020 | https://guidedoc.com/best-superfoods-list |
| 21 | Bing | 2022 | https://artofhealthyliving.com/best-superfoods-to-boost-your-healthy-diet |
| 22 | Bing | 2021 | https://www.healthygreensavvy.com/top-superfoods |
| 23 | Bing | 2021 | https://www.cosmopolitan.com/health-fitness/a38581530/superfoods-list-2022 |
| 24 | Bing | 2022 | https://blog.mindvalley.com/best-superfoods/ |
| 25 | Bing | 2022 | https://formnutrition.com/inform/the-a-to-z-of-superfoods |
| 26 | Bing | 2022 | https://www.olivemagazine.com/recipes/healthy/15-best-superfood-recipes |
| 27 | Bing | 2021 | https://www.medicinenet.com/what_are_the_10_best_foods_to_eat/article.htm |
| 28 | Bing | 2022 | https://www.healthline.com/nutrition/50-super-healthy-foods |
| 29 | Bing | 2021 | https://www.hollandandbarrett.com/the-health-hub/conditions/immunity/immunity-nutrition/immune-system-supporting-foods |

^1^ Entry in ‘People also ask’ section of Google search engine.

**Table S2.** List of selected web pages found in the second page of search engines.

| **Order No.** | **Search engine** | **Year** | **URL** |
| --- | --- | --- | --- |
| 30 | Google | 2022 | https://www.nebraskamed.com/primary-care/how-superfoods-work-plus-a-list-of-best-superfoods |
| 31 | Google | 2021 | https://www.ucdavis.edu/food/what-makes-superfood-so-super |
| 32 | Google | 2021 | https://www.mysportscience.com/post/the-myth-of-superfoods |
| 33 | Google | 2022 | https://www.realsimple.com/health/nutrition-diet/healthy-eating/top-superfoods-according-to-dietitians |
| 34 | Google | 2021 | https://draxe.com/nutrition/what-are-superfoods/ |
| 35 | Google | 2022 | https://www.healthshots.com/healthy-eating/superfoods/what-are-superfoods-and-why-are-they-important-for-healthy-diet/ |
| 36 | Google | 2022 | https://www.today.com/health/diet-fitness/superfoods-improve-brain-power-boost-energy-rcna24795 |
| 37 | Google | 2022 | https://www.floridatoday.com/story/life/wellness/2022/05/03/here-10-superfoods-sure-boost-your-immunity-make-you-feel-better/7445000001/ |
| 38 | Google | 2022 | https://www.cnbc.com/2022/03/05/doctor-shares-best-superfoods-she-includes-in-her-diet-for-a-strong-healthy-immune-system.html |
| 39 | Google | 2022 | https://www.houstonchronicle.com/lifestyle/renew-houston/nutrition/article/Are-superfoods-real-Be-cautious-of-labels-that-16989075.php |
| 40 | Google | 2022 | https://www.masterclass.com/articles/superfoods-explained#7-types-of-superfoods |
| 41 | Bing | 2022 | https://www.hsph.harvard.edu/nutritionsource/superfoods |
| 42 | Startpage | 2022 | https://www.heart.org/en/healthy-living/healthy-eating/eat-smart/nutrition-basics/get-smart-about-superfoods-infographic |
| 43 | Startpage | 2020 | https://www.self.com/gallery/20-superfoods-slideshow |
| 44 | Startpage | 2021 | https://www.eatthis.com/best-ever-weight-loss-superfoods/ |
| 45 | Startpage | 2022 | https://www.mindbodygreen.com/articles/superfoods-to-enhance-your-vitality-from-energy-specialist |
